# The effect of mitochondrial transplantation therapy from different gender on inhibiting cell proliferation of malignant melanoma

**DOI:** 10.7150/ijbs.59581

**Published:** 2021-05-11

**Authors:** Zhenyao Yu, Yixue Hou, Wei Zhou, Zizhen Zhao, Zesheng Liu, Ailing Fu

**Affiliations:** School of Pharmaceutical Sciences, Southwest University, Chongqing 400715, China.

**Keywords:** mitochondrial transplantation therapy, melanoma, cell cycle arrest, apoptosis

## Abstract

Today mitochondria are considered much more than a energy plant in cells. Mitochondrial transplantation therapy has been an active research area for treating mitochondria-associated diseases from animal studies to clinical trials. However, the specific mechanism involved in the anti-tumor activity of healthy mitochondria remain to be characterized. Here we investigate the signal mechanism and gender difference of mitochondrial transplantation therapy against malignant melanoma. In the study, we administrated intact mitochondria extracted from mouse livers respectively to the mice bearing malignantly subcutaneous and metastatic melanoma, and identified the signal mechanism responsible for the mitochondrial treatment through transcriptomic analysis. Meanwhile, the efficiency of female mitochondria and male mitochondria was compared in the cultured melanoma cells and transplanted melanoma in mice. The results suggested that the mitochondria significantly inhibited the tumor cell proliferation *in vitro* through cell cycle arrest and induction of cell apoptosis. In the melanoma-bearing mice, the mitochondria retard the tumor growth and lung migration, and the transcriptomic analysis indicated that general chromosome silencing was strongly associated with the mitochondria against melanoma after the mitochondrial transplantation on the metastasis melanoma. Moreover, the anti-tumor activity of mitochondria from female animals was more efficient in comparison to the males, and the female mitochondria could probably induce more persuasive mitochondria-nuclear communication than the mitochondria from male mice. The study identifies the anti-tumor mechanism of the mitochondrial transplantation therapy, and provides a novel insight into the effect of mitochondria from different gender.

## Introduction

Mitochondria are the crucial organelle that is responsible for cell survival and apoptosis. Healthy mitochondria are essential to maintain the normal function of cells. However, accumulating research evidence identifies that tumor mitochondria undergo adaptive changes to accelerate rapid proliferation of tumor cells in the acidic and hypoxic microenvironment 1. Thus, introducing healthy mitochondria into tumor cells is proposed to have a high efficacy in preventing tumor growth 2,3. Currently, exogenous healthy mitochondria have been utilized in treating several carcinoma, including breast cancer, pancreatic cancer, and glioma, and similar results exhibited the excellent anti-tumor activity of the healthy mitochondria 4-7. Although biochemical measurement after mitochondrial transplantation suggested that healthy mitochondria can significantly decrease the oxidative phosphorylation (OXPHOS) capability and induce apoptosis in the tumor cells. However, the molecular signal mechanism for the mitochondrial transplantation therapy is still unclear.

Moreover, mitochondria are the near-exclusive maternal inheritance in evolution, and there might be sex differences of mitochondria in transplantation therapy. The earlier findings reported that mitochondria from female animals (female mitochondria) are more sensitive to stress and are better equipped to deal with the harmful condition 8,9, thereby the female mitochondria were assumed to have different activities in anti-tumor growth in comparison with the male mitochondria. In this study, we investigated both signal mechanism and gender difference of the mitochondrial transplantation therapy. The study would not only clarify the molecular signal of the mitochondria on the tumor but also provide a new insight of the anti-tumor effect of mitochondria obtained from different gender.

## Results

### Mitochondrial characteristics from male and female mice

**Figure 1:**
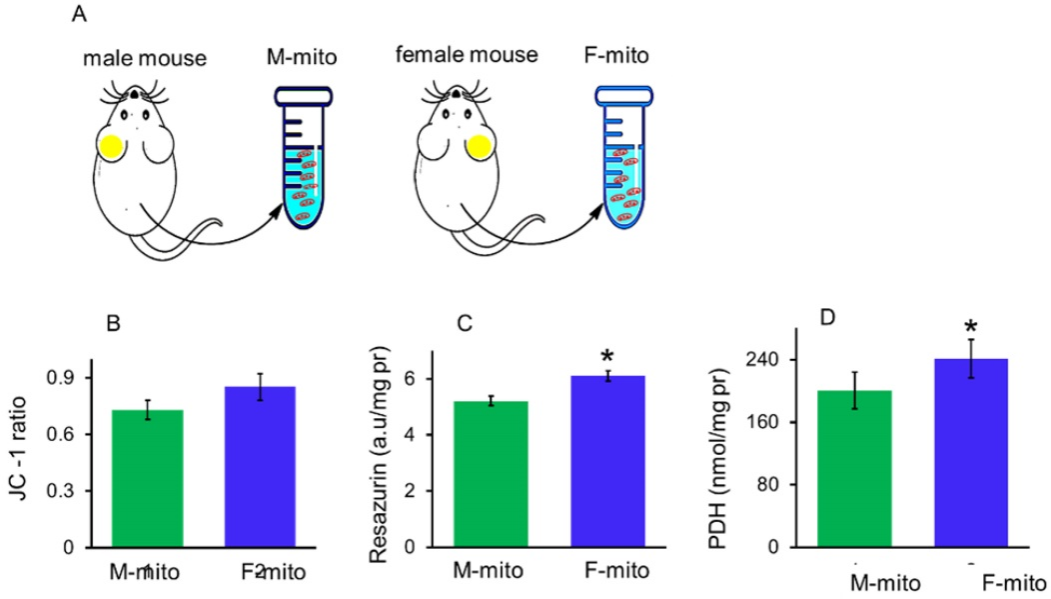
The activity of mitochondria isolated from the male and female mice. (A), A schematic diagram of female and male mitochondria isolated from mice. M-mito, mitochondria from male mice; F-mito, mitochondria from female mice. The activity of both mitochondria were respectively measured by using methods of JC-1 (B), resazurin (C), and PDH (D). The data were expressed as mean ± SD (n = 6). *p < 0.05 compared with the M-mito group.

Mitochondrial membrane potential, redox ability and pyruvate dehydrogenase (PDH) activity can be used to indicate membrane integrity and activity of mitochondria. In addition, PDH is one of key enzymes of OXPHOS, and one of its subunit PDHA1 locates on the X chromosome, region p22.1 in somatic tissues 10. In a previous report, mitochondria of females have higher protein content and ATP production capacity than males 11. Thus itochondrial membrane potential, redox ability, and PDH activity were measured respectively to identify the difference between the mitochondria from male mice (M-mito) and female mice (F-mito) (Fig. 1A), Increasing trends of mitochondrial membrane potential and redox ability were showed in F-mito compared with M-mito despite of without significant difference between the two groups (Fig. 1B). Nevertheless, PDH activity of F-mito was higher than those of M-mito (Fig. 1C and 1D).

### Mitochondria prevented melanoma cell proliferation

**Figure 2:**
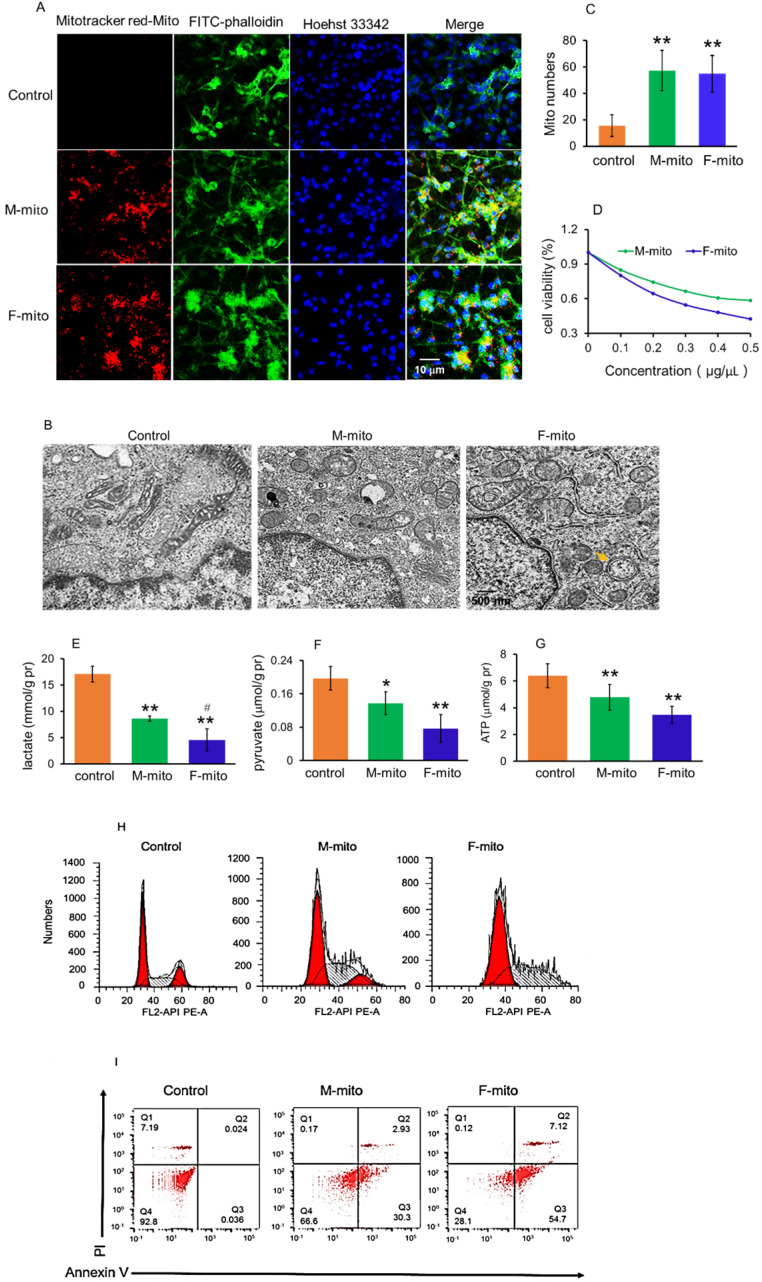
Mitochondria inhibited melanoma cell proliferation. (A) Mitochondria could enter cells. The mitochondria were labeled by Mitotracker Red CMXRos, and cytoskeletons and nucleus were respectively stained by FITC-phalloidin and Hoechst 33342. M-mito, mitochondria from male mice; F-mito, mitochondria from female mice. (B), Mitochondrial morphology was observed under TEM. Yellow arrow pointed to the autophagic body. (C) The numbers of mitochondria in each cell section. The numbers were counted under TEM. The data were expressed as mean ± SD (n = 15). **p < 0.01 compared with control (without mitochondrial treatment). (D), cell viability decreased in a concentration-dependent manner after M-mito and F-mito treatment. (E-G), Contents of lactate, pyruvate, and ATP decreased after the respective addition of M-mito and F-mito into the cells. The data were expressed as mean ± SD (n = 6). *p < 0.05, **p < 0.01 compared with control. ^#^*p* < 0.05 compared with the M-mito-treated group. (H)The cell cycle was arrested by mitochondria. (I) Cell apoptosis was measured by flow cytometry. The melanoma cells treated by M-mito and F-mito treatment respectively were collected and stained by Annexin V-FITC and PI for cell apoptosis assay.

The cell internalization of mitochondria in melanoma cells was evaluated by using the confocal microscopy, where mitochondria were labeled by commercial MitoTracker Red CMXRos, and the cell skeleton was stained by FITC-phalloidin. After incubation with mitochondria for 2 h, a prominent yellow fluorescence was identified in the melanoma cells (Fig. 2A), as shown by overlay of the fluorescence of MitoTracker red and FITC. Moreover, the morphology of mitochondria in the cells was observed by using TEM, and the images showed that the mitochondria in melanoma cells exhibited elongated and cavitating shape (Fig. 2B). In contrast, these in mitochondria-treated cells represented a spherical and rod-like structure. Also, autophagic bodies appeared in the mitochondria-treated cells. Under the TEM, the mitochondrial numbers significantly increased after mitochondrial addition, assessed by counting the mitochondrial numbers on each image (Fig. 2C).

To determine the capacity of the mitochondria on melanoma, we examined the effect of the mitochondria on cell viability in anoxic conditions using the anaerobic incubator. As illustrated in Fig. 2D, mitochondrial treatment for 24 h resulted in reduction of cell viability in a concentration-dependent manner, and F-mito-treated cells had lower viability than the cells treated by M-mito.

Since tumor cells produce energy through aerobic glycolysis (Warburg Effect) under hypoxia condition 12,13, we examined the levels of pyruvate and lactate, the metabolites of glycolysis, as well as ATP content after the mitochondria were introduced into the melanoma cell media. The results exhibited that both pyruvate and lactate levels were reduced, accompanying with decreases of ATP content in the mitochondria-treated cells (Fig. 2E-2G). In addition, F-mito showed a more substantial inhibitory effect in tumor cell proliferation than M-mito, evidenced by lower levels of pyruvate, lactate, and ATP in the F-mito-treated cells than those of M-mito-treated cells. There was significant difference in lactate level between the F-mito-treated cells and the M-mito-treated cells, but no significant difference exhibited in the levels of pyruvate and ATP between the cells (Fig. 2E-2G).

Moreover, cell cycle and apoptosis were examined by using flow cytometry. The cell cycle was prominently arrested after mitochondrial addition (Fig. 2H), and approximately 33.2% in M-mito-treated cells and 71.8% in F-mito-treated cells showed apoptosis after mitochondrial treatment (Fig. 2I), proving the mechanism through which mitochondria inhibit cell proliferation could be in relation with the initiation of cell cycle arrest and cell apoptosis in the melanoma cells.

### Mitochondria inhibited the growth of subcutaneous melanoma

**Figure 3:**
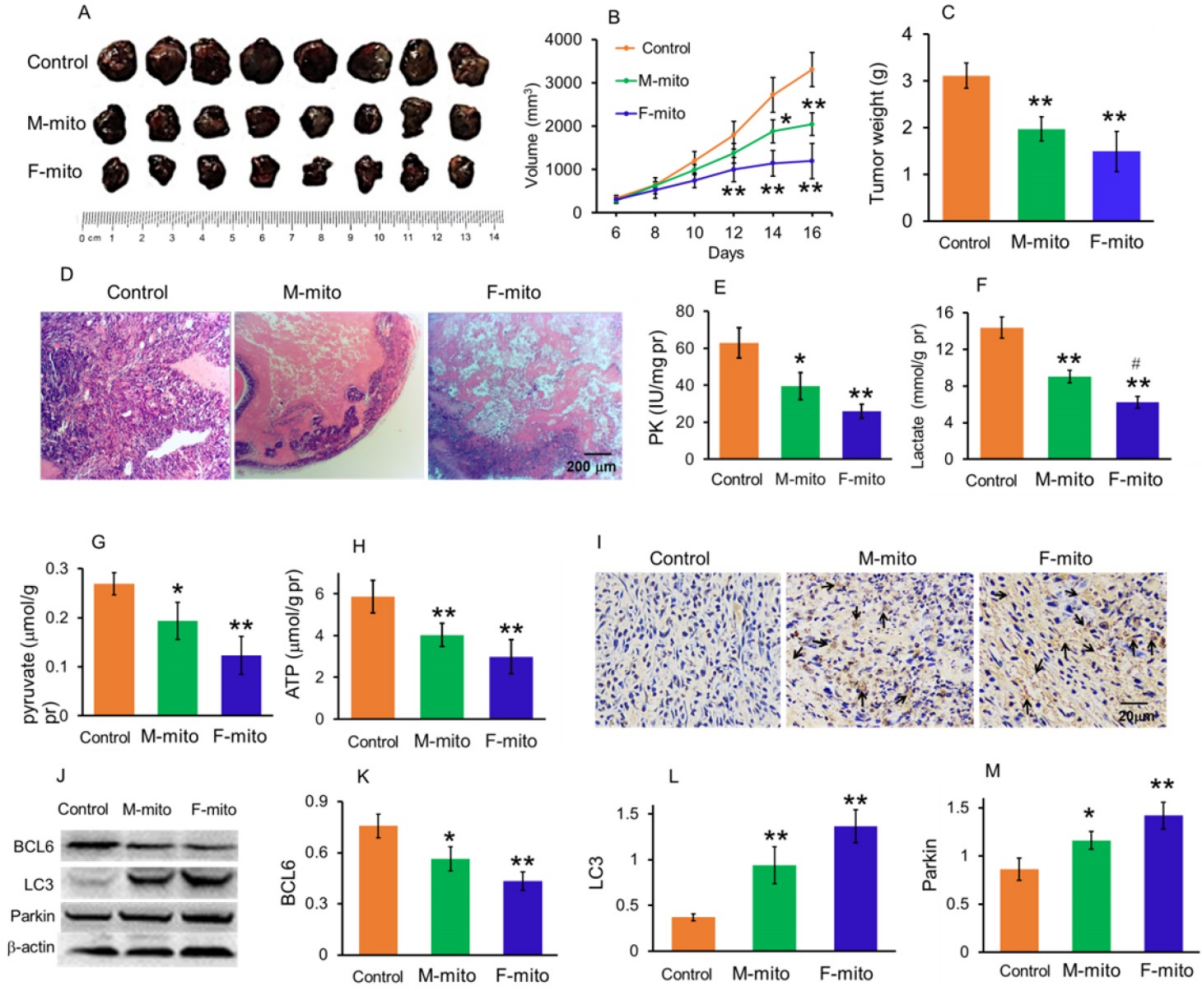
Mitochondria inhibited the growth of subcutaneous melanoma. (A), melanoma separated from the subcutaneous tissues. M-mito, mitochondria from male mice; F-mito, mitochondria from female mice. (B) The growth curve of tumor volume in each group. The data were expressed as the mean ± SD (n = 8). *p < 0.05, **p < 0.01 compared with the model control group. (C), melanoma weight of each group at 16 days after 10^7^ cell transplantation. (D), HE staining of melanoma. (E-H), the levels of PK, lactate, pyruvate, and ATP decreased after M-mito and F-mito administration. N = 6 for each group.^ #^*p* < 0.05 compared with the M-mito-treated group. (I), Cell apoptosis was detected by TUNEL staining. The arrows pointed to the apoptotic cells. (J), levels of BCL6, LC3, and Parkin were respectively detected by western blot. (K-M), ratios of gray values BCL6, LC3, and Parkin in Western blot analysis.

To prepare model of subcutaneous melanoma, mouse melanoma B16 cell line was transplanted subcutaneously 14,15. After the B16 cell transplantation, the volume of subcutaneous tumors in the model group increased continuously. Then the melanoma-bearing mice were randomly divided into 3 groups as model group, female mitochondria-treated group (F-mito), and male mitochondria-treated group (M-mito). The isolated mitochondria (10^6^) were injected intravenously once a day in the mitochondria-treated group. When the mice were euthanized at 16th days after transplantation, the tumor volume was 3307.7 ± 395.2 mm^3^ (Fig. 3A and 3B), and the weight reached about 3.1 g (Fig. 3C). However, the growth rate of tumors slowed down after the mitochondrion administration. On the 5th day after intravenous injection of F-mito, the tumor volume began to shrink, and the growth rate slowed down. After the 11^th^ day of F-mito injection, the tumor volume reduced to 1198.8 ± 408.5 mm^3,^ and the weight was about 1.5 g. Also, after intravenous injection of M-mito for 11 days, the tumor volume was 2042.1 ± 260.9 mm^3^, and the weight was about 2.0 g.

On the 16^th^ day of cell transplantation, the subcutaneous melanomas were respectively removed out and stained by HE. The images showed that the tissues in the model group were full of tumor cells (Fig. 3D), whereas cell numbers reduced and gaps appeared in the mitochondria-treated groups. In addition, the results of biochemical measurement showed that the PK, one of the key enzymes of glycolysis, as well as the contents of lactate and pyruvate, significantly reduced. Accordingly, the ATP level was also significantly decreased. In addition, the levels of PK, lactate, pyruvate, and ATP in the F-mito-treated group were lower than those in the M-mito-treated group.

**Figure 4:**
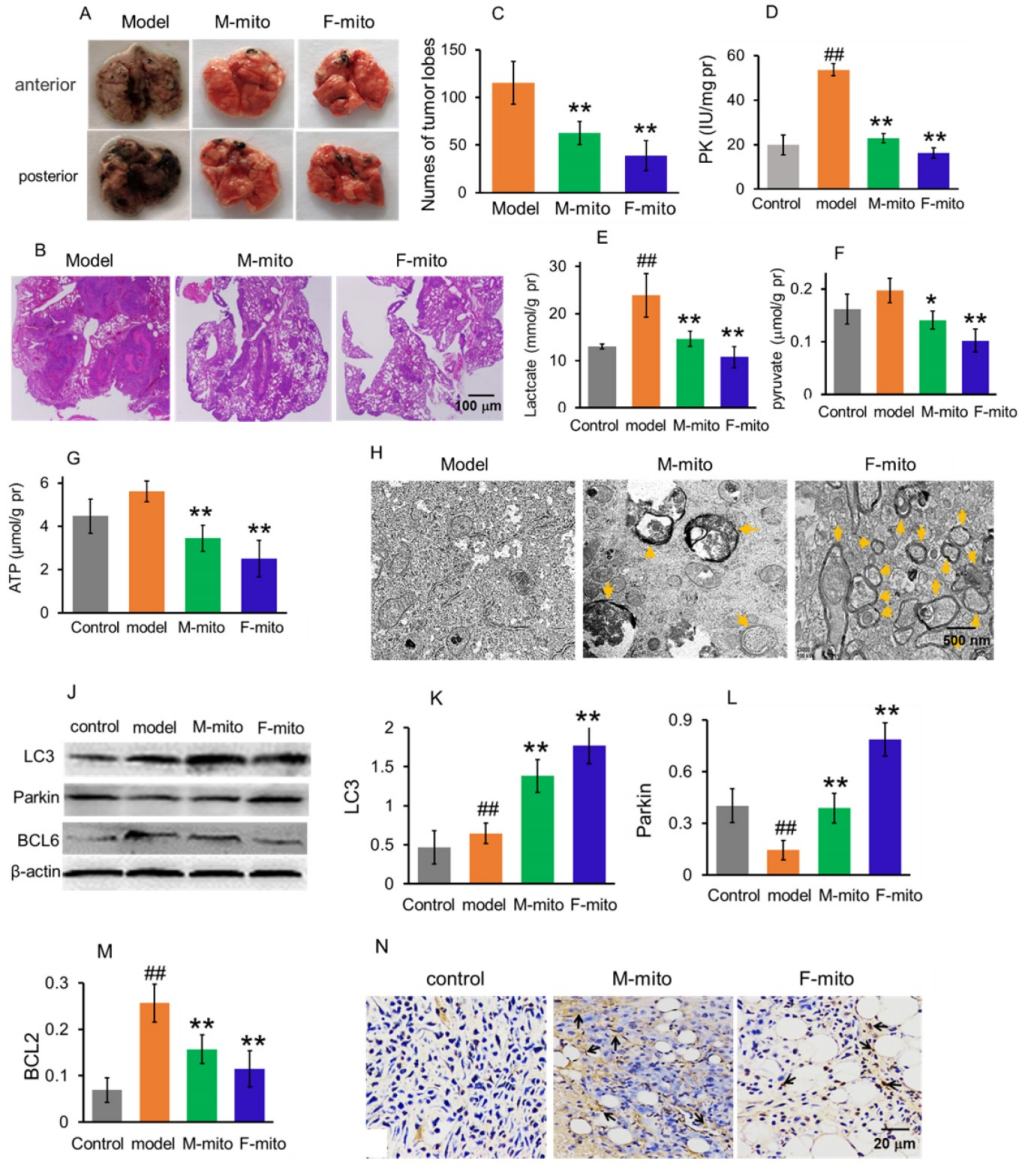
Mitochondria inhibited the growth of metastatic melanoma in the lungs. (A), representative pictures of lungs in each group at 24 days after mitochondrial administration. M-mito, mitochondria from male mice; F-mito, mitochondria from female mice. (B) HE staining showed melanoma nodules in lung tissues. (C), nodule numbers of melanoma of each group. The data were expressed as the mean ± SD (n = 8). **p < 0.01 compared with the model group. (D-G), The levels of PK, lactate, pyruvate, and ATP were examined in the melanoma. N = 6 for each group. ^##^*p* < 0.01 compared with the control group. *p < 0.05, **p < 0.01 with the model group. (H), Mitochondria of melanoma tissues were observed under TEM. Yellow arrows pointed to autophagy. (J), Western blot of LC3, parkin, and BCL6 proteins in each group. (K-M), ratios of the gray value of western blot. N = 6 for each group. ^##^*p* < 0.05 compared with the normal control; *p < 0.05, **p < 0.01 with the model group. (N) Cell apoptosis was detected by TUNEL staining. The arrows pointed to the apoptotic cells.

Moreover, TUNEL staining was carried out to determine apoptosis after mitochondrial injection since mitochondria were a key player in cell apoptosis. As shown in Fig. 4I, numbers of TUNEL-positive cells in melanoma tissues were stained brown by DAB in the mitochondria-treated mice. Also, tumor cells in the F-mito-treated group had enormous TUNEL-positive cells than those of the M-mito-treated group.

To further identify the cell apoptosis induced by mitochondrial administration, Western blot was performed to examine the level of BCL2, an anti-apoptotic protein that increased in tumor cell proliferation (Fig. 3J). The gray value showed that the mitochondria could significantly decrease the BCL2 level (Fig. 3K), suggesting that the anti-apoptotic ability of tumor cells might be inhibited. In addition, the levels of autophagy-related proteins, LC3, and parkin, remarkably increased in melanoma cells after mitochondrial administration, especially in the F-mito-treated group (Fig. 3J, 3L, and 3M).

### Mitochondria prevented the proliferation of metastatic lung melanoma

Since one of the leading causes of mortality in melanoma is lung metastasis, here the animal model of metastatic lung melanoma was prepared to examine the therapeutic effect of the mitochondria. The mouse melanoma B16 cells were injected intravenously to prepare the model 7. After the cell transplantation, the numbers of melanoma nodules increased daily, and the lung color changed to dark in the model mice. The model mice at autopsy exhibited large and dense nodules on day 16 after cell transplantation, calculated through gross anatomy and HE staining technique (Fig. 4A and 4B). However, tumor development was prevented after mitochondrial treatment, showing smaller and lower tumor nodules in the lungs (Fig. 4A-C). The suppressive effect of melanoma is more prominent in the F-mito-treated group. Then levels of PK, lactate, pyruvate, and ATP were respectively measured to identify the metabolic changes after mitochondrial administration. Similarly, with the results of mitochondrial action on subcutaneous melanoma, the mitochondria decreased the levels of PK, lactate, pyruvate, and ATP (Fig. 4D-G), suggesting that energy production through glycolysis was inhibited in the metastatic melanoma cells.

Moreover, mitochondria in the melanoma were visualized under the TEM. Only a few numbers of mitochondria were visualized in the model group, whereas in the mitochondrial treatment group, the number of mitochondria increased predominantly. Also, mitochondria appeared as vacuoles and with swelling, and there were also autophagosomes in the melanoma cells of the treatment group (Fig. 4H), which could be related to the increase of protein levels of LC3 and parkin (autophagy-related proteins) (Fig. 4J-L). Besides, the expression of BCL2 protein decreased after mitochondrial treatment (Fig. 4J and 4M). Meanwhile, the images of TUNEL staining showed that a large number of TUNEL-positive cells appeared in the mitochondria-treated group (Fig. 4N), indicating that mitochondria could initiate cell apoptosis through reduction of the anti-apoptotic protein.

### Transcriptomic analysis of metastatic melanoma after mitochondrial treatment

In order to elucidate the molecular mechanism of mitochondrial inhibition on melanoma, transcriptome analysis was used to identify the genes and molecular signals involved in mitochondrial transplantation. Analysis results revealed the difference of DEGs in metastatic melanoma between the model group and mitochondrial treatment group was about 2.7 fold changes. A quantity of 509 DEGs were statistically categorized from the gathered transcripts, involving 137 up-regulated and 372 down-regulated DEGs genes (Fig. 6A). The operational role of these DEGs was explained by reference to the Genome of Mus_musculus.GRCm38.dna.primary_assembly.fa.gz (Gene build by Ensembl), and also complemented with the explanation of homologous data collected by utilizing a blastx search kit versus public protein database, namely eggNOG, UniProtID, and Kyoto Encyclopedia of Genes and Genomes (KEGG). Then gene ontology (GO) enhancement was conducted to establish the DEGs' operational classification. The DEGs genes were classified into three main classes, comprising cellular constituent, molecular function, and biological process. The cellular constituent cluster was further divided into 22 subclasses, molecular function into 34 subclasses, and biological process into 47 subclasses. The most represented GO terms in the biological process category were the mitotic cell cycle, cell division, chromosome segregation, sister chromatid segregation, mitotic nuclear division, and nuclear chromosome segregation. The subclasses of the chromosome, centromeric region, condensed chromosome, centromeric region, kinetochore, and spindle were the most profuse GO terms in the cellular constituent category. For the molecular function category, the most terms were anion binding, ion binding, ATP binding, and DNA replication origin binding.

**Figure 5:**
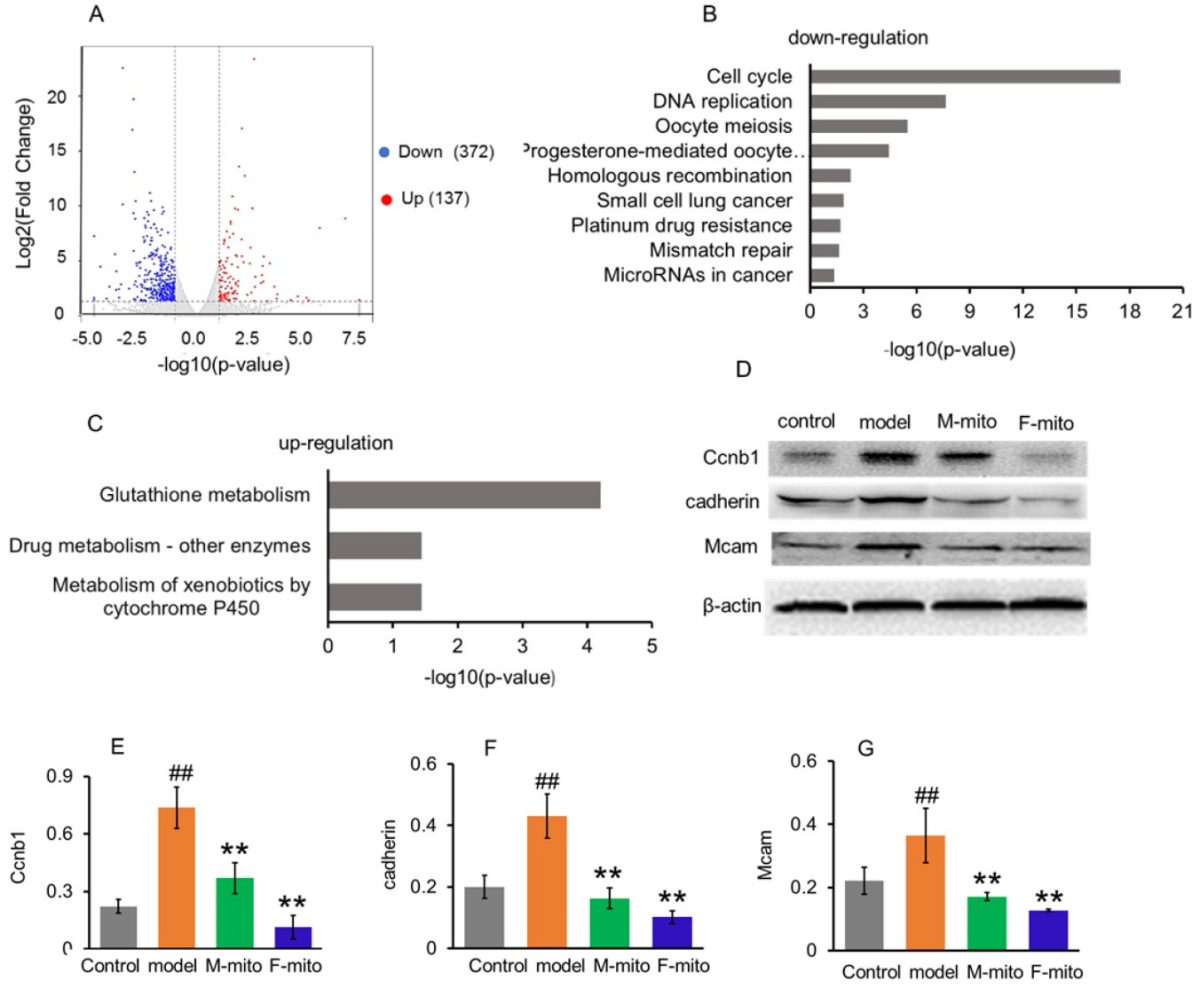
Go analysis and Enriched KEGG pathways after mitochondrial administration. (A), a summary of the numbers of up- and down-regulated DEGs for GO subcategory. (B), the down-regulated 9 significantly enriched KEGG pathways, and (C), the up-regulated 3 KEGG pathways. The pathways were generalized according to *p* < 0.05. (D), Western blot analysis of reprehensive protein expression of DEGs. M-mito, mitochondria from male mice; F-mito, mitochondria from female mice. (E-G), Ratios of Western blot bands of Ccnb1, cadherin, and Mcam to β-actin. N = 6 for each group. ^##^*p* < 0.05 compared with the control; **p < 0.01 with the model group.

To further clarify the molecular signals, the DEGs were sorted into the canonical pathways through the analysis of KEGG cellular pathways. A sum of 219 DEGs (46 up-regulated and 173 down-regulated genes) was categorized into 27 statistically consequential classes. Beyond the *p* < 0.05, the KEGG-annotated DEGs were predominantly refined into six main pathways: cellular processes (4), environmental information processing (1), genetic information processing (4), human diseases (7), metabolism (11), and organismal systems (3). The majority of down-regulated KEGG pathways were closely associated with cancer cell proliferation, including cell cycle, DNA replication, and mitosis (Fig. 5B), while three up-regulated pathways were involved in glutathione metabolism, drug and xenobiotic metabolism (Fig. 5C).

### Mitochondria inhibited gene transcription of the tumor cell proliferation

From the KEGG pathway, the cell cycle was significantly arrested by the mitochondria. An extensive range of genes involved in the cell cycle and division were down-regulated after mitochondrial treatment (Table S1). The result was consistent with the Western blot analysis of CCNB1 (one of representative cell cycle regulators) (Fig. 5D and 5E), and with the melanoma cell cycle arrest after mitochondrial administration by using flow cytometry (Fig. 2B).

Meanwhile, mitochondrial transplantation therapy inhibited the cancer-related gene transcription in melanoma. As tumorigenesis and development, numbers of cancer-related genes are transcribed and expressed to maintain the unlimited proliferation of tumor cells. However, a series of the genes were down-regulated after mitochondrial treatment, including *Cadherin*, *Nid1*, *Hmmr*, *Rrm2*, *Raet1e*, *Nipal1*, *Birc5*, *Iqgap3*, *Ckap2*, *Golm1*, *Myom2*, *Kif20a*, *Dlgap5*, *Afp*, *Ect2*, *Mcam*, etc. (Table S2). Also, several genes of cancer suppressors were activated after mitochondrial administration, including *Csad*, *Ccdc30*, *Macrod1*, *Pcdh1*, *Tprkb*, and *Hdac11* (Table S2). Among proteins expressed by these genes, cell adhesion molecules cadherin and Mcam are suggested to represent melanoma progression markers, which involve melanoma progression, proliferation, migration, and invasion 16. However, mitochondrial transplantation therapy remarkably reduced the protein expressions of cadherin and Mcam, identified by Western blot (Fig. 5D, 5F, and 5G).

### Mitochondria regulated the gene transcription of autophagy- and apoptosis-related proteins in the melanoma

The result of DEG analysis exhibited that the mitochondria activated gene transcription of autophagy-related proteins (*Gabarapl1*, *LC3*, *Atg*, *Prkn*, *Pink1*) (Table S3). The activation would lead to an increase of the autophagy protein expression (Fig. 2B and 4H) and the emergence of autophagy (Fig. 3J and 4J). Moreover, mitochondria down-regulated gene transcription of anti-apoptotic proteins, including *BCL6*, *Naip6*. *Naip5*, *Ddias*, *Apaf1,* and *Stk17b*, but increased the transcription of mitochondrion-associated apoptosis-inducing factor gene (*Aifm3*) (Table S4). As a consequence, it appeared numbers of apoptotic cells in the melanoma after treated by the mitochondria, evaluated by flow cytometry and TUNEL staining (Fig. 2E, 3I, and 4N).

### Molecular signal pathway of mitochondrial transplantation therapy on melanoma

In order to indentify all the relevant cell signaling pathways produced by the mitochondria, we outlined the signaling regulatory network through the existing DNA-protein interaction database and protein-protein interplay (Cytoscape 3.5.0 software). Gene regulation by chromosome silencing and mitochondrial unfold protein reaction (UPR^mt^) induced by mitochondrial stress was considered to be the most relevant mechanism in the series of biological activities of the mitochondrial transplantation therapy. The overall gene silencing of tumor cell proliferation caused by the mitochondria would be related to methylation modification of histone. Meanwhile, the mitochondrial stress would promote the local opening of UPR^mt^ regulatory region to induce gene transcription of tumor suppressor proteins and autophagy-related proteins. The signal pathways could explain the signal mechanism of the inhibitory effect of mitochondria on melanoma.

## Discussion

Currently, melanoma has become the leading cause of death from skin disease 17. Many efforts have been made to find ways to treat the terrible disease. Here we suggest that the tumor growth of both subcutaneous and metastatic malignant melanoma was significantly inhibited after mitochondrial administration. The signal mechanism was closely associated with the chromosome silencing and the local UPR^mt^ effect caused by mitochondrial stress, eventually leading to cell cycle arrest, energy deficiency, autophagy, and cell apoptosis. The tumor inhibition effect of F-mito in cell cycle arrest and energy depletion was more potent than that of M-mito.

Mitochondria play a crucial role in tumor cell signal transduction 18. The proliferation of tumor relies heavily on mitochondrial function, which can rapidly and profoundly affect the molecular signal transmission in cells 19,20. Due to unlimited rapid proliferation, the tumor cells lie in the anoxic and acidic microenvironment, then the function of native mitochondrial of tumor cells has changed to adapt to this hostile environment 21. In melanoma, mitochondrial DNA (mtDNA) deletion and mutation are common features. In a study of 67 melanoma patients, 4977 bp and 5128 bp deletions in mtDNA are found in melanoma tissues induced by solar radiation 22. Also, mtDNA mutations are active contributors in ultraviolet-radiation- independent melanoma carcinogenesis 23. Moreover, the dysfunctional mitochondria accumulate through nonselective fusion due to the WNT/β-catenin signaling activation in melanoma, leading to mitochondrial remodeling and then rapid proferation and invasion of the melanoma cells 24. However, the microenvironment would cause the dysfunction of healthy mitochondria and then lead to mitochondrial stress when the healthy mitochondria arrive in the tumor cells.

**Figure 6:**
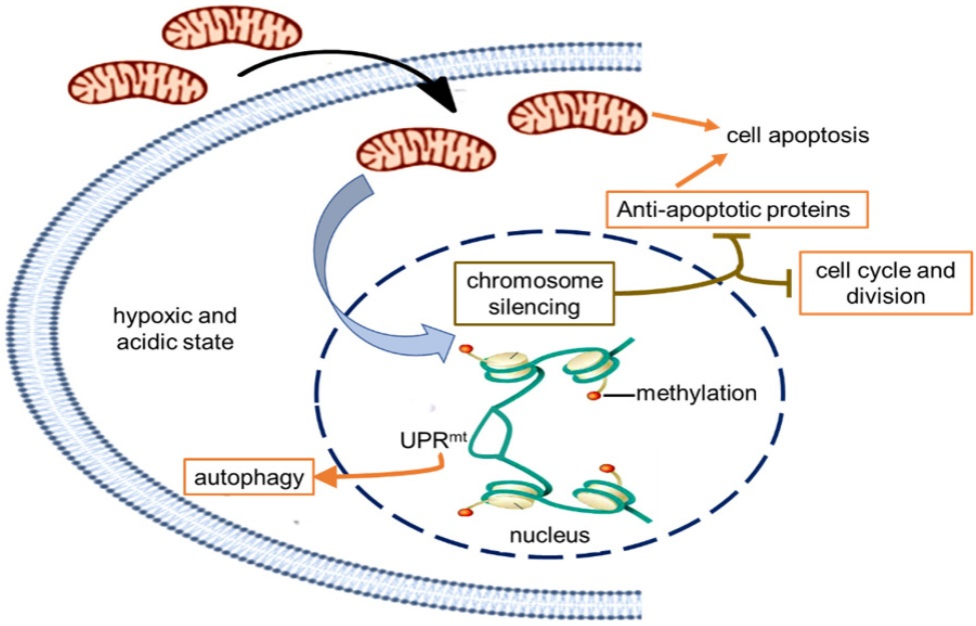
Schematic diagram of the possible mechanism of tumor growth inhibition by treating with mitochondria. The healthy mitochondria would incur stress within hypoxic and acidic tumor environment, whereas adaptive changes have taken place in native mitochondria to maintain the unlimited proliferation of tumor cells. Then the mitochondrial stress could promote chromosome silencing through histone methylation modification, resulting in cell cycle arrest and down-regulation of tumor activating genes. As the gene expression of anti-apoptotic proteins was inhibited, mitochondria would activate the apoptosis pathway. However, only in the UPR^mt^ effect region, the portion of the chromosome was opened up, and the expression of UPR^mt^-induced proteins increased.

Retrograde transcriptional signaling of mitochondria plays vital role as stress sensors to adapt its environment 25. The retrograde signals between mitochondria and nucleus, including ATP, ROS, UPR^mt^, 2-hydroxyglutarate, opening of mitochondrial permeability transition pore, can regulate gene expression in the nucleus 26,27. Recently, a potential link between mitochondrial stress and widespread changes in chromatin structure has been proposed 28. Mitochondrial stress response activates chromatin reorganization through histone H3K9 di-methylation that is associated with gene silencing 29. Changes in epigenome caused by mitochondrial stress are usually expansive and enduring 30,31, resulting in tumor cell proliferation stagnation (Fig. 6).

Nevertheless, portions of the chromatin can open up to modulate cellular responses to stress even if the chromatin globally becomes silenced. Among signals mediated by mitochondrial stress, UPR^mt^ is a potential mitochondria-nuclear retrograde pathway that has an essential impact on cell function through regulating the expression of nuclear genes 32,33,34. Activated UPR^mt^ can induce the up-regulation of glutathione metabolism, autophagy, and tumor suppressor 35. The autophagy in an anoxic environment and tumor suppressor expression could cause tumor growth inhibited.

Moreover, mitochondria are key players in cell apoptosis 36. Healthy mitochondria usually induce cell apoptosis under pathological hypoxia to eliminate damaged cells. However, tumor cells down-regulate apoptotic signaling of mitochondria, particularly the activation of anti-apoptotic systems, allows the cells to escape from apoptosis leading to uncontrolled proliferation in the tumor microenvironment 37. In the study, the gene transcription of anti-apoptotic proteins was generally down-regulated that might be related to chromosome silencing after mitochondrial administration, then the cell apoptosis could be induced to eradicate tumor cells.

In order to determine which mitochondria from different genders have a stronger effect on the future application in animals, we tested the sex differences of mitochondria on treating melanoma. Although numbers of reports described tissue-specific sex differences in mitochondrial morphology and oxidative capacities 38,39, scarcely studies showed the functional differences of mitochondria in therapy. From the limited data available, female mitochondria have more favorable mitochondria-nuclear communication in response to stress compared to male mitochondria 40. In this study, we firstly assessed mitochondrial activities isolated from female and male mice, and the results showed that female mitochondria exhibited higher activity and ATP production ability than those of male mitochondria. Subsequently, the anti-tumor experiments both *in vitro* and *in vivo* proved that female mitochondria have higher efficacy to inhibit tumor cell proliferation than male mitochondria. The study also revealed that female mitochondria could induce more robust stress response to silence gene transcription than male mitochondria in tumor cells, suggesting that female mitochondria were more sensitive under the hypoxic microenvironment of the tumor than male mitochondria, and eventually led to the stronger anti-tumor effect.

Here we used intact mitochondria to study their anti-tumor activity through the intravenous administration. After the mitochondria are injected into blood vessels, they would increase the content of mitochondria in blood. It is known that blood contains intact cell-free mitochondria in both healthy people and cancer patients, but the level of circular mitochondrial DNA in plasma of the cancer patients is proportionally lower than that of normal individuals 41,42, implying that sufficient mitochondrial content in blood would be important in maintainiing normal physiological functions. The entry mechanism of mitochondria into cells would be associated with macropinocytosis-mediated endocytosis because macropinocytosis inhibitor can prevent the internalization of mitochondria by cells 43,44. Moreover, mitochondria have been condidered as systemic messengers between cell-cell communication 45, and meanwhile, mitochondria can be uptaken into various types of cells by *in vitro* and *in vivo* studies 46,47. Thus, we assumed that the mitochondria in blood could be internalized by melanoma cells because of the high permeability of local tumor blood vessels (enhanced permeability and retention effect) 48, which was evidenced by TEM of melanoma tissues (Fig. 4). Further, mitochondria in blood can activate immune system through increasing the activities of phagocytes and T cells 49,50, which might increase the anti-tumor effect of the mitochondria to a certain extent. The mitochondria on tumor immunity will be further investigated.

In summary, mitochondria are important signal organelles in cells, which closely regulate the tendency of survival and death of cells. This study exhibits a novel insight into mitochondrial function in malignant melanoma, and propose that healthy mitochondria inhibit tumor cell proliferation by preventing tumor gene transcription. The overall down-regulation of genes results in cell cycle arrest and cell proliferation stagnation, as well as the activation of autophagy and apoptosis, which eventually leads to the apparent inhibition of melanoma growth after mitochondrial transplantation therapy. The effect of female mitochondria on anti-melanoma is stronger than that of male mitochondria, which may explain why incidence rate and mortality rate of melanoma in female are lower than those in male. The study not only reveals the anti-tumor mechanism of the mitochondria transplantation but also suggests female mitochondria have more potent than male mitochondria on anti-melanoma.

## Materials and Methods

### Animals

Healthy BABL/c mice of 2 month-age-old (22 ± 2 g) were used in the research. The mice were procured from the Chongqing Medical University, China. The mice were housed in SPF center and fed by standard mouse chow and water. The animal protocol was authenticated through the Animal Ethical Committee, Southwest University.

### Mitochondrial isolation and activity measurement

Liver mitochondria from female and male mice were isolated according to the earlier reported protocol 51. Further, the mice were euthanized quickly through cervical dislocation, and the mouse liver was isolated and homogenized at 4 °C. The supernatant fraction was collected, and an isolated mitochondrial solution was kept for homogenization. The redox capacity of the isolated mitochondria was examined using resazurin, and membrane potential were obtained by JC-1 assay kits procured from Jiangsu Kaiji Biotech. Ltd. Co, Nanjing, China. The activity of pyruvate dehydrogenase (PDH) was measured according to the manufacturer's protocol (Nanjing Jiancheng Biotech. Institute, Nanjing, China). Each measurement was independently conducted about six times, respectively.

### Cell culture and viability tests

Murine melanoma B16 cells were cultured with RPMI 1640 media (containing 10% FBS) in a hypoxic incubator chamber with 37 °C, 5% CO_2_. When the tumor cell mass reached 60% in 96-well plates, the isolated mitochondria were respectively added into the wells. The cells were observed using a confocal microscope (Zeiss. Germany) after mitochondrial addition. After 24 h incubation with the mitochondria, cell viability was measured by Alamar blue according to the manufacturer protocol. The levels of lactate, pyrvate, and ATP in cells were respectively determined using the commercial kits obtained from Nanjing Jiancheng Biotech. Institute, Nanjing, China. The mitochondrial morphology in tumor cells was examined using the transmission electron microscope (TEM). Further, cell cycle and apoptosis were respectively measured by using flow cytometery (BD FACS Calibur, USA).

### Preparation of subcutaneous melanoma mouse model

In order to prepare the mouse model of subcutaneous melanoma, the melanoma B16 cell suspension (cell concentration 10^7^/mL) with a volume of 0.2 mL was subcutaneously injected into the subaxillary of the right forelimb. After 6 days of cell transplantion, the longest diameter (L) and the largest transverse diameter (W) of the vertical direction of tumor mass were measured by using the vernier caliper, respectively. The volume of the tumor (V) was calculated according to the formula: V = LW^2^/2.

### Animal assignment and mitochondrial administration

After 6 days of cell transplantation, the mice were randomly classified into 3 groups. The groups were assigned as tumor model group, female mitochondria group (F-mito), and male mitochondria group (M-mito). In the mitochondria treating group, the isolated exogenous mitochondria (10^6^) were injected intravenously once in a day, while the mice in the model group were injected with the same amount of saline. Every group contained 8 mice (n = 8 in each group) for the mitochondrial therapy on the subcutaneous melanoma. The subcutaneous tumors in each group were observed and recorded until the mice in the model group were euthanized through the administration of overdosage of sodium pentobarbital. Then the tumor tissues were isolated and weighed, and stained with HE. Meanwhile, the tissues were homogenated to respectively measure the levels of pyruvate kinase (PK), lactate, pyruvate, and ATP according to the commercial kits (Nanjing Jiancheng Biotech. Institute, Nanjing, China).

### Preparation of mouse pulmonary metastatic melanoma and mitochondrial treatment

The tumor cells in the logarithmic growth period were digested by 0.25% trysin/EDTA and adjusted the concentration to 10^6^/mL. Then the cell suspension (0.2 mL) was administered into the tail vein. The mice were given mitochondrial therapy once a day at the sixth day after cell transplantation. On the 24^th^ day, the mice were euthanized, and the numbers of metastatic tumor colonies per lung were examined. Subsequently, the melanoma mass was respectively removed out for further measurement.

### Western blot

Protein extraction of melanoma was exposed to SDS-PAGE and transmitted onto PVDF membrane, further investigated with antibodies versus BCL6, LC3, parkin, Ccnb, Cadherin, Mcam and β-actin (Beijing boason Biotech. Co., Ltd., Beijing, China), then with HRP-conjugated secondary antibodies (1:5000; Beijing Dingguo Biotech. Co., Ltd., Beijing, China) as the secondary antibody. Further, the membranes were thoroughly washed twice for about 15 min for each wash, and the signals were respectively recognized by the ECL system (Pierce Co., USA).

### Immunohistochemistry for cell apoptosis

TUNEL (terminal deoxynucleotidyl transferase-mediated nick end labeling) staining was conducted on the tumor tissues obtained from the mice. Apoptotic cells were identified by using DAB in-situ cell apoptosis kit according to the manufacturer's protocol (Beijing Beyotime Biotec. Co. Beijing, China). Tissues sections were observed under an optical microscope (Olympus, Japan).

### Transcriptomic analysis

In order to elucidate the molecular mechanism of mitotherapy on the tumor, the metastatic lung melanomas of each group were dissected for transcriptomic analysis. In short, the total RNA of the melanomas was extracted based on the manufacturer's procedure, and then NanoDrop ND-2000 (Thermo Scientific, USA) was used to examine the purity and quantification of the total RNA. The library construction and sequencing were carried out by Shanghai Personalbio Biotechnology Co., Ltd. (Shanghai, China). Differential expression genes (DEGs) between model and mitotherapy groups were identified through the calculation of the gene expression level of each transcript according to the fragments per kilobase of exon per million mapped reads (FRKM) method. Then SEM and edgeR software were respectively used to measure the concentration of genes/isoform and differential expression analysis. For the functional annotations, the collected transcripts were searched using String, KEGG databases, and NCBI protein nonredundant (NR) databases. For Gene Ontology (GO) annotations, the BLAST2GO program and KEGG database were used to analyze the pathways.

### Statistical analysis

Data were expressed in terms of mean ± SD. Differences between control and mitochondria-treated group were assessed by using two-way ANOVA and least-significant differences (LSD) for post hoc comparisons (*p* < 0.05). Differences between F-mito- and M-mito-treated group were assessed using One-way ANOVA and Student's t-test to compare statistical significance (*p* < 0.05). For transcriptomic analysis, the mean difference criteria of the transcription between groups are set as [log2FoldChange] > 1 and *p* < 0.05. All statistical analyses were performed with SPSS 13.0 for Windows software.

## Supplementary Material

Supplementary tables.

## References

[1] Jing X, Yang F, Shao C, Wei K, Xie M, Shen H (2019). Role of hypoxia in cancer therapy by regulating the tumor microenvironment. Mol Cancer.

[2] Singh B, Modica-Napolitano JS, Singh KK (2017). Defining the momiome: promiscuous information transfer by mobile mitochondria and the mitochondrial genome. Semin Cancer Biol Semin Cancer Biol.

[3] Roushandeh AM, Kuwahara Y, Roudkenar MH (2019). Mitochondrial transplantation as a potential and novel master key for treatment of various incurable diseases. Cytotechnology.

[4] Chang JC, Chang HS, Wu YC, Cheng WL, Lin TT, Chang HJ (2019). Mitochondrial transplantation regulates antitumour activity, chemoresistance and mitochondrial dynamics in breast cancer. J Exp Clin Cancer Res.

[5] Sun C, Liu X, Wang B, Wang Z, Liu Y, Di C (2019). Endocytosis-mediated mitochondrial transplantation: Transferring normal human astrocytic mitochondria into glioma cells rescues aerobic respiration and enhances radiosensitivity. Theranostics.

[6] McCully JD, Levitsky SE, Nido PJ, Cowan DB (2016). Mitochondrial transplantation for therapeutic use. Clin Transl Med.

[7] Fu A, Hou Y, Yu Z, Zhao Z, Liu X (2019). Healthy mitochondria inhibit the metastatic melanoma in lungs. Int J Biol Sci.

[8] Justo R, Boada J, Frontera M, Oliver J, Bermúdez J, Gianotti M (2005). Gender dimorphism in rat liver mitochondrial oxidative metabolism and biogenesis. Am J Physiol Cell Physiol.

[9] Ventura-Clapier R, Moulin M, Piquereau J, Lemaire C, Mericskay M, Veksler V (2017). Mitochondria: a central target for sex differences in pathologies. Clin Sci (Lond).

[10] Dahl H, Hutchison WM, Zheng G, Forrest SM, Hansen LL (1991). Polymorphisms in the human X-linked pyruvate dehydrogenase Ela gene. Hum Genet.

[11] Justo R, Boada J, Frontera M, Oliver J, Bermúdez J, Gianotti M (2005). Gender dimorphism in rat liver mitochondrial oxidative metabolism and biogenesis. Am J Physiol Cell Physiol.

[12] Wang Y, Bai C, Ruan Y, Liu M, Chu Q, Qiu L (2019). Coordinative metabolism of glutamine carbon and nitrogen in proliferating cancer cells under hypoxia. Nat Commun.

[13] Zhu X, Xuan Z, Chen J, Li Z, Zheng S, Song P (2020). How DNA methylation affects the Warburg effect. Int J Biol Sci.

[14] Lu L, Zhang J, Gan P, Wu L, Zhang X, Peng C (2021). Novel functions of CD147 in the mitochondria exacerbates melanoma metastasis. Int J Biol Sci.

[15] Wang Q, Fu C, Li X, Hou Y, Fu A (2019). Mechanism of melanoma growth inhibition by exogenous mitochondria. Acta Pharm Sin.

[16] Watson-Hurst K, Becker D (2006). The role of N-Cadherin, MCAM, and β3 integrin in melanoma progression, proliferation, migration and invasion. Cancer Biol Ther.

[17] Owens B (2014). Melanoma. Nature.

[18] Qureshi MA, Haynes CM, Pellegrino MW (2017). The mitochondrial unfolded protein response: Signaling from the powerhouse. J Biol Chem.

[19] Yoshida GJ (2015). Metabolic reprogramming: the emerging concept and associated therapeutic strategies. J Exp Clin Cancer Res.

[20] Vyas S, Zaganjor E, Haigis MC Mitochondria and cancer. 2016; 166: 555-566.

[21] Avnet S, Baldini N, Brisson L, De Milito A, Otto AM, Pastoreková S (2018). Annual meeting of the international society of cancer metabolism (ISCaM): cancer metabolism. Front Oncol.

[22] Hubbard K, Steinberg ML, Hill H, Orlow I (2008). Mitochondrial DNA deletions in skin from melanoma patients. Ethn Dis.

[23] Mitra D, Luo X, Morgan A, Wang J, Hoang MP, Lo J (2012). An ultraviolet-radiation-independent pathway to melanoma carcinogenesis in the red hair/fair skin background. Nature.

[24] Brown K, Yang P, Salvador D, Kulikauskas R, Ruohola-Baker H, Robitaille AM (2017). WNT/β-catenin signaling regulates mitochondrial activity to alter the oncogenic potential of melanoma in a PTEN-dependent manner. Oncogene.

[25] Vyas S, Zaganjor E, Haigis MC (2016). Mitochondria and Cancer. Cell.

[26] Zhou Q, Zhu L, Qiu W, Liu Y, Yang F, Chen W, Xu R (2020). Nicotinamide riboside enhances mitochondrial proteostasis and adult neurogenesis through activation of mitochondrial unfolded protein response signaling in the brain of ALS SOD1G93A mice. Int J Biol Sci.

[27] Zhao X, Ma X, Guo J, Mi M, Wang K, Zhang C (2019). Circular RNA circEZH2 suppresses transmissible gastroenteritis coronavirus-induced opening of mitochondrial permeability transition pore via targeting miR-22 in IPEC-J2. Int J Biol Sci.

[28] Merkwirth C, Jovaisaite V, Durieux J, Matilainen O, Jordan SD, Quiros PM (2016). Two conserved histone demethylases regulate mitochondrial stress-induced longevity. Cell.

[29] Tian Y, Garcia G, Bian Q, Steffen KK, Joe L, Wolff S (2016). Mitochondrial Stress Induces Chromatin Reorganization to Promote Longevity and UPR(mt). Cell.

[30] Mayorga L, Salassa BN, Marzese DM, Loos MA, Eiroa HD, Lubieniecki F (2019). Mitochondrial stress triggers a pro-survival response through epigenetic modifications of nuclear DNA. Cell Mol Life Sci.

[31] Tatar M, Sedivy JM (2016). Mitochondria: masters of epigenetics. Cell.

[32] Kenny TC, Germain D (2017). mtDNA, mtastasis, and the mtochondrial unfolded protein response (UPRmt). Front Cell Dev Biol.

[33] Aldridge JE, Horibe T, Hoogenraad NJ (2007). Discovery of genes activated by the mitochondrial unfolded protein response (mtUPR) and cognate promoter elements. PLoS One.

[34] Siegelin MD, Dohi T, Raskett CM, Orlowski GM, Powers CM, Gilbert CA (2011). Exploiting the mitochondrial unfolded protein response for cancer therapy in mice and human cells. J Clin Invest.

[35] Munch C, Harper JW (2016). Mitochondrial unfolded protein response controls matrix pre-RNA processing and translation. Nature.

[36] Holley AK, Dhar SK, Clair D (2010). Manganese superoxide dismutase versus p53: the mitochondrial center. Ann N Y Acad. Sci.

[37] Mohammada RM, Muqbila I, Lowec L, Yedjoud C, Hsue HY, Linf LT (2015). Broad targeting of resistance to apoptosis in cancer. Semin Cancer Biol.

[38] Ventura-Clapier R, Moulin M, Piquereau J, Lemaire C, Mericskay M, Veksler V (2017). Mitochondria: a central target for sex differences in pathologies. Clin Sci (Lond).

[39] Beaudry KM, Devries MC (2019). Sex-based differences in hepatic and skeletal muscle triglyceride storage and metabolism. Appl Physiol Nutr Metab.

[40] Demarest TG, McCarthy MM (2015). Sex differences in mitochondrial (dys)function: Implications for neuroprotection. J Bioenerg Biomembr.

[41] Meddeb R, Dache ZAA, Thezenas S, Otandault A, Tanos R, Pastor B (2019). Quantifying circulating cell-free DNA in humans. Sci Rep.

[42] Al Amir Dache Z, Otandault A, Tanos R, Pastor B, Meddeb R, Sanchez C (2020). Blood contains circulating cell-free respiratory competent mitochondria. FASEB J.

[43] Kitani T, Kami D, Matoba S, Gojo S (2014). Internalization of isolated functional mitochondria: involvement of macropinocytosis. J Cell Mol Med.

[44] Zhao Z, Hou Y, Zhou W, Keerthiga R, Fu A (2020). Mitochondrial transplantation therapy inhibit CCl4-induced liver injury through scavenging free radicals and protecting hepatocytes. Bioeng Transl Med.

[45] Torralba D, Baixauli F, Sánchez-Madrid F (2016). Mitochondria know no boundaries: mechanisms and functions of intercellular mitochondrial transfer. Front Cell Dev. Biol.

[46] Fu A (2020). Mitotherapy as a novel therapeutic strategy for mitochondrial diseases. Curr Mol Pharmacol.

[47] Patel D, Rorbach J, Downes K, Szukszto MJ, Pekalski ML, Minczuk M (2017). Macropinocytic entry of isolated mitochondria in epidermal growth factor-activated human osteosarcoma cells. Sci Rep.

[48] Maeda H, Tsukigawa K, Fang J (2016). A retrospective 30 years after discovery of the enhanced permeability and retention effect of solid tumors: next-generation chemotherapeutics and photodynamic therapy-problems, solutions, and prospects. Microcirculation.

[49] Zhao Z, Yu Z, Hou Y, Zhang L, Fu A (2020). Improvement of cognitive and motor performance with mitotherapy in aged mice. Int J Biol Sci.

[50] Desdín-Micó G, Soto-Heredero G, Aranda JF, Oller J, Carrasco E, Gabandé-Rodríguez E (2020). T cells with dysfunctional mitochondria induce multimorbidity and premature senescence. Science.

[51] Shi XX, Bai HY, Zhao M, Li XR, Sun XC, Jiang HB (2018). Treatment of acetaminophen-induced liver injury with exogenous mitochondria in mice. Transl Res.

