# Supplementary material for: The effect of mitochondrial transplantation therapy from different gender on inhibiting cell proliferation of malignant melanoma
Source: Int J Biol Sci. 2021 May 11;17(8):2021–33. doi: 10.7150/ijbs.59581 (PMC8193273; doi:10.7150/ijbs.59581)
Supplement: Supplementary file 1 — Supplementary tables. [file ijbsv17p2021s1.pdf]

Supplementary materials

Table S1. Mitochondria down-regulated cell cycle-associated gene transcription.

**gene**	**log2foldchange**	**p-value**	**EC**	**description**
Cdca3	-2.07	3.56E-11	-	cell division cycle associated 3
Pbk	-2.77	3.74E-11	EC:2.7.12.2	PDZ binding kinase
Cdc6	-3.34	7.34E-11	-	cell division cycle 6
Cdc20	-1.95	2.44E-10	-	cell division cycle 20
Ccnb1	-2.25	3.61E-10	-	cyclin B1
Mcm6	-2.86	4.6E-10	EC:3.6.4.12	minichromosome maintenance complex component 6
Ccna2	-1.94	4.99E-09	-	cyclin A2
Plk1	-2.31	9.51E-09	EC:2.7.11.21	polo like kinase 1
Ccnb2	-1.84	1.14E-08	-	cyclin B2
Nuf2	-2.25	5.57E-08	-	NUF2, NDC80 kinetochore complex component
Bub1	-2.42	7.15E-08	EC:2.7.11.1	BUB1, mitotic checkpoint serine/threonine kinase
Top2a	-1.86	1.12E-06	EC:5.6.2.2	topoisomerase (DNA) II alpha
Kntc1	-2.86	1.25E-06	-	kinetochore associated 1
Smc2	-1.42	3.43E-06	-	structural maintenance of chromosomes 2
Cdca2	-2.06	3.71E-06	-	cell division cycle associated 2
Knl1	-2.16	4.66E-06	-	kinetochore scaffold 1

Cip2a	-1.73	4.88E-06	-	cell proliferation regulating inhibitor of proteinphosphatase 2A
Chek1	-2.73	1.63E-05	EC:2.7.11.1	checkpoint kinase 1
Prim1	-1.59	1.72E-05	EC:2.7.7.10 2	DNA primase, p49 subunit
Chek1	-2.73	1.63E-05	EC:2.7.11.1	checkpoint kinase 1
Knstrn	-1.47	2.11E-05	-	kinetochore-localized astrin/SPAG5 binding
Ccnd1	-1.04	3.93E-05	-	cyclin D1
Pif1	-2.56	4.52E-05	EC:3.6.4.12	PIF1 5'-to-3' DNA helicase
Mcm7	-1.13	4.85E-05	EC:3.6.4.12	minichromosome maintenance complex component 7
Tlr8	-1.36	5.21E-05	-	toll-like receptor 8
Enpp2	-0.95	5.49E-05	EC:3.1.4.39	ectonucleotide pyrophosphatase/phosphodiesterase 2
Cdk1	-1.64	7.66E-05	-	cyclin-dependent kinase 1
Inca1	1.19	1.17E-04	-	inhibitor of CDK, cyclin A1 interacting protein 1
Nusap1	-1.67	6.07E-04	-	nucleolar and spindle associated protein 1
Lig1	-1.57	7.38E-04	-	ligase I, DNA, ATP-dependent
Cenpa	-1.01	7.65E-04	-	centromere protein A
Cdc45	-1.73	1.27E-03	-	cell division cycle 45
Topbp1	-0.92	1.46E-03	-	topoisomerase (DNA) II binding protein 1
Tceal5	-1.71	1.84E-03	-	transcription elongation factor A (SII)-like 5

G0s2	0.79	2.79E-03	-	G0/G1 switch gene 2
Ccne2	-1.16	3.49E-03	-	cyclin E2
Cdca5	-1.66	5.91E-03	-	cell division cycle associated 5
Cdca8	-1.09	1.13E-02	-	cell division cycle associated 8
Cdc25c	-1.83	1.38E-02	EC:3.1.3.48	cell division cycle 25C
Polk	-1.02	1.66E-02	EC:2.7.7.7	polymerase (DNA directed), kappa
Ska3	-1.67	1.81E-02	-	spindle and kinetochore associated complex subunit3
Ccng1	-0.57	1.86E-02	-	cyclin G1
Chek2	-0.95	2.46E-02	EC:2.7.11.1	checkpoint kinase 2
Orc1	-2.51	3.36E-02	-	origin recognition complex, subunit 1
Ccne1	-0.78	3.77E-02	-	cyclin E1
Map4k4	-0.64	4.27E-02	EC:2.7.11.1	mitogen-activated protein kinase

Table S2. Mitochondria regulated cancer-associated proteins.

**gene**	**log2fold change**	**p-value**	**EC**	**description**
Csad	2.56	4.23E-24	EC:4.1.1.29	cysteine sulfinic acid decarboxylase
Nid1	-1.55	1.33E-10	-	nidogen 1
Hmmr	-2.18	6.61E-10	-	hyaluronan mediated motility receptor
Rrm2	-2.38	1.30E-09	EC:1.17.4.1	ribonucleotide reductase M2

Raet1e	-2.37	1.31E-09	-	retinoic acid early transcript 1E
Nipal1	-2.21	2.48E-09	-	NIPA-like domain containing 1
Ccdc30	6.68	1.42E-09	-	coiled-coil domain containing 30
Birc5	-1.92	4.46E-09	-	baculoviral IAP repeat-containing 5
Iqgap3	-2.34	6.17E-09	-	IQ motif containing GTPase activating protein 3
Golm1	-1.59	1.45E-08	-	golgi membrane protein 1
Myom2	-1.24	5.70E-08	-	myomesin 2
Macrod1	1.46	7.41E-08	-	MACRO domain containing 1
Kif20a	-2.07	1.46E-07	-	kinesin family member 20A
Dlgap5	-2.37	1.98E-07	-	DLG associated protein 5
Afp	-2.12	3.37E-07	-	alpha fetoprotein
Ect2	-1.56	4.08E-07	-	ect2 oncogene
Gpnmb	-1.18	6.18E-07	-	glycoprotein (transmembrane) nmb
Stmn1	-1.34	1.95E-06	-	stathmin 1
Cdh17	-3.70	2.54E-06	-	cadherin 17
Tpx2	-1.83	2.81E-06	-	TPX2, microtubule-associated
Lox	-2.14	3.28E-06	EC:1.4.3.13	lysyl oxidase
Ube2c	-1.63	3.63E-06	EC:2.3.2.23	ubiquitin-conjugating enzyme E2C
Clspn	-2.62	1.48E-05	-	claspin
Anln	-1.57	2.50E-05	-	anillin, actin binding protein

Trp53inp1	-0.98	3.87E-04	-	transformation related protein 53 inducible nuclearprotein 1
Tekt5	-2.36	5.69E-04	-	tektin 5
Pcdh1	0.81	7.18E-04	-	protocadherin 1
Tprkb	0.79	1.00E-03	-	Tp53rk binding protein
Tmem229a	-1.38	2.28E-03	-	transmembrane protein 229A
Rnase4	1.42	7.67E-03	EC:3.1.27.5	ribonuclease, RNase A family 4
Msn	-0.70	1.12E-02	-	moesin
Coro1c	-0.64	1.13E-02	-	coronin, actin binding protein 1C
Itm2a	1.24	1.18E-02	-	integral membrane protein 2A
Hdac11	0.67	1.22E-02	EC:3.5.1.98	histone deacetylase 11
Bcam	-0.78	1.28E-02	-	basal cell adhesion molecule
Cdh3	-1.68	1.57E-02	-	cadherin 3
Mcam	-0.39	2.02E-02	-	melanoma cell adhesion molecule
Cadm1	-0.58	2.34E-02	-	cell adhesion molecule 1
Casc4	-0.81	2.97E-02	-	cancer susceptibility candidate 4
Maged2	-0.61	3.17E-02	-	melanoma antigen, family D, 2
Itgbl1	-0.85	4.91E-02	-	integrin, beta-like 1

Table S3. Mitochondria activated autophagy-associated gene transcription.

**gene**	**log2fold change**	**p-value**	**EC**	**description**

Gabarapl1	0.84	3.33E-04	-	gamma-aminobutyric acid (GABA) A receptor-associated protein-like 1
Map1lc3a	0.89	8.39E-04	-	microtubule-associated protein 1 light chain 3alpha
Atg5	0.40	1.08E-02	-	autophagy related 5
Prkn	0.84	4.01E-02	EC:2.3.2.31	parkin RBR E3 ubiquitin protein ligase
Pink1	0.72	4.92E-02	EC:2.7.11.1	PTEN induced putative kinase 1

Table S4. Mitochondria regulated gene transcription of apoptosis-related proteins.

**gene**	**log2fold change**	**p-value**	**EC**	**description**
BCL6	-1.96	2.14E-06	-	B cell leukemia/lymphoma 6
Naip6	-1.17	5.94E-03	-	NLR family, apoptosis inhibitory protein 6
Naip5	-1.05	8.36E-03	-	NLR family, apoptosis inhibitory protein 5
Aifm3	0.92	1.51E-02	EC:2.7.12.2	apoptosis-inducing factor, mitochondrion-associated3
Ddias	-1.57	2.36E-02	-	DNA damage-induced apoptosis suppressor
Apaf1	-0.63	4.12E-02	-	apoptotic peptidase activating factor 1
Stk17b	-0.54	4.28E-02	EC:2.7.11.1	serine/threonine kinase 17b (apoptosis-inducing)
